# AI agents in Alzheimer’s disease management: challenges and future directions

**DOI:** 10.3389/fnagi.2025.1735892

**Published:** 2026-01-05

**Authors:** Gerasimos Grammenos, Aristidis G. Vrahatis, Konstantinos Lazaros, Themis P. Exarchos, Panagiotis Vlamos, Marios G. Krokidis

**Affiliations:** 1 Bioinformatics and Human Electrophysiology Laboratory, Department of Informatics, Ionian University, Corfu, Greece; 2 Institute of Digital Biomedicine, Ionian University Research and Innovation Center, Corfu, Greece

**Keywords:** Alzheimer’s disease, autonomous AI agents, clinical decision, explainable AI, large language models

## Abstract

Neurodegenerative diseases such as Alzheimer’s and Parkinson’s disease pose a major global healthcare challenge, with cases projected to rise sharply as populations age and effective treatments remain limited. AI has shown promise in supporting diagnostics, predicting disease progression, and exploring biomarkers, yet most current tools are narrowly focused, unimodal, and lack longitudinal reasoning or interpretability. By enabling context-aware analysis across imaging, genomics, cognitive, and behavioral data, agentic AI can track disease progression, identify therapeutic targets, and support clinical decision-making. Over time, these systems may detect gaps in their own information and request targeted data, moving closer to real clinical reasoning while keeping clinicians in control. The next frontier in medical AI lies in developing autonomous, multimodal agents capable of integrating diverse data, adapting through experience, supporting decision-making, and collaborating with clinicians. Furthermore, ethical, patient-centered AI requires close technical-clinical collaboration to support clinicians and improve patient outcomes. This perspective examines AI’s current role in Alzheimer’s care, identifies key challenges in integration, interpretability, and regulation, and explores pathways for safely deploying these agentic systems in clinical practice.

## Introduction

Neurodegenerative diseases such as Alzheimer’s related dementias and Parkinson’s disease represent one of the greatest challenges in global healthcare. According to World Alzheimer’s Report over 55 million people live with dementia, mostly due to Alzheimer’s worldwide while this number is projected to reach 150 million people worldwide by 2050 (Alzheimer’s Disease International, 2024). As human lifespan increases, more individuals suffer cognitive decline or motor dysfunction while effective treatments and reliable early diagnostics remain limited. This growing gap between disease burden and therapeutic progress highlights the need for new ways to understand and manage these complex clinical. Artificial intelligence (AI) has shown progress in this area as a supportive tool in diagnostic classification, disease prediction, biomarker discover and disease mechanism exploration (van Oostveen and de Lange, 2021). For example, AI analysis of neuroimaging biomarkers can assist in early prediction of Alzheimer’s disease (AD) pathology (Kale et al., 2024). Table 1 presents an overview of recent AI applications in the context of AD management. This illustrates AI’s supportive role in neurodegenerative disease research, from diagnosing early symptoms to predicting which patients with mild cognitive impairment will progress to dementia (Ai et al., 2025). However, most current artificial intelligence tools in medicine remain narrowly focused, with strong performance in isolated tasks such as MRI classification or speech analysis but limited capacity to generalize well using multimodal data. Recent evidence confirms that clinical AI systems largely remain unimodal and lack mechanisms for integrating heterogeneous data or supporting longitudinal reasoning (Winchester et al., 2023; Judge et al., 2024). They operate as passive algorithms rather than active collaborators and frequently function as black boxes with limited interpretability (Rudin, 2019; Xu and Shuttleworth, 2024). Herein, we argue that the frontier in medical AI is to move beyond narrow, task-specific models toward autonomous agents that can reason across varied data sources, use multiple existing state-of-the-art tools for decision support and calculation, adapt through experience and collaborate effectively with clinicians. We specifically examine the current role of AI in AD’s care, outline key obstacles related to integration, interpretability and regulation, considering future directions for introducing these agents into clinical practice.

**TABLE 1:** Recent AI applications in the context of Alzheimer’s disease management.

Research area	Methodology	Modality	Outcome	References
Diagnostic classification	Deep learning with latent space analysis	Structural MRI	Classifies sex-specific brain network changes with high precision (> 90%).	Wang et al., 2024
	Multimodal data fusion	MRI + plasma biomarkers	Detects amyloid status with AUC 0.94	Jasodanand et al., 2025
	EEG/electrophysiological AI models	EEG recording	Identification of the EEG frequency bands decisive for AD diagnosis, balance metrics, real-time AD detection	Arikan et al., 2025
Disease prediction	Knowledge-graph learning	EHR	Predicts AD onset 7 years in advance (AUC 0.80).	Tang et al., 2024
	Trajectory modeling	Longitudinal MRI + CSF (Aβ42)	Forecasts MCI-to-AD conversion within 3 years with 85% accuracy.	Agostinho et al., 2024
Biomarker discovery	Feature selection and machine learning classifiers	Blood gene expression from ADNI, ANM1, ANM2 datasets	Classification of AD (AUC: 0.619–0.859); identification of AD-related pathways (inflammation, mitochondria, Wnt signaling)	Lee and Lee, 2020
	Classical machine learning	Digital biomarkers: motor activity, neurocognitive tests, eye tracking, speech analysis	Prediction/classification of AD (AUC ∼0.887) and MCI (AUC ∼0.821); integration into clinical practice	Qi et al., 2025
Mechanism exploration	GS correlation analysis; imaging-transcriptomics integration	fMRI; gene expression	Altered GS topography in AD; correlation with functional network properties and cognition	Chen et al., 2023
	Deep transfer learning using pre-trained CNNs, ensemble modeling	MRI	Accurate AD diagnosis (up to 96% accuracy) with interpretable visual explanations highlighting key neural regions	Mahmud et al., 2024

## Clinical landscape of Alzheimer’s disease

AD is a progressive neurodegenerative disorder clinically characterized by the cognitive impairment and decline in various functional abilities. Several processes are involved into the pathophysiology of AD, i.e., abnormal extracellular deposition of amyloid-beta (Aβ) plaques, intracellular tau tangles, and volumetric loss of cortical gray matter (DeTure and Dickson, 2019). This pathophysiology is already present long before the clinical AD onset symptoms, which is known as “preclinical” AD and it can last for up to 20 years. The commonest clinical manifestation of the AD is the short-term memory deficits, while modest impairments in other cognitive domains can also be present, known as “prodromal AD” or Mild Cognitive Impairment (MCI) stage. During the disease course cognitive decline is worsening leading to dementia, a syndromic term for a decline in cognitive abilities of sufficient severity to interfere with function during daily activities.

Available pharmacological treatments provide only temporary symptomatic relief and do not alter disease course. Non-pharmacological interventions, such as cognitive training, physical activity, nutrition, and social engagement show proven benefits, but remain fragmented, non-standardized, and rarely integrated with biological or digital monitoring (Luo et al., 2023). Current care models therefore fail to deliver adaptive and personalized interventions capable of addressing the heterogeneity of AD. Traditional biomarkers (CSF assays, PET, plasma NfL) enable early diagnosis and stratification while digital biomarkers derived from wearables and smartphones offer scalable, real-time measures of cognition and behavior. Evidence from meta-analyses supports combined cognitive-behavioral strategies, which also have transdiagnostic relevance (e.g., depression, psychosis) ( Ó hAnrachtaigh et al., 2024). However, integration of digital signals into clinical workflows is minimal and combined biological–digital biomarker frameworks for guiding interventions are lacking. Recent advances in AI provide new opportunities to link multimodal biomarkers with clinical outcomes, but these remain at early research stages and are not embedded in routine dementia care (Jasodanand et al., 2025).

## The growing importance of agentic AI

Agentic AI (artificial intelligence), refers to intelligent systems capable of acting autonomously, perceiving their surroundings, reasoning about what they observe, and making decisions to achieve specific objectives. Unlike conventional software that follows fixed instructions, agentic systems are designed to learn from experience, plan their actions, and adapt dynamically as conditions change. They combine perception, reasoning, memory and action into a coherent whole, allowing them to operate with a level of independence that brings machines closer to genuine collaboration with humans. This development marks a significant step in artificial intelligence, as systems evolve from static automation to entities capable of purposeful and context-aware behavior (Huang, 2025).

**FIGURE 1:**
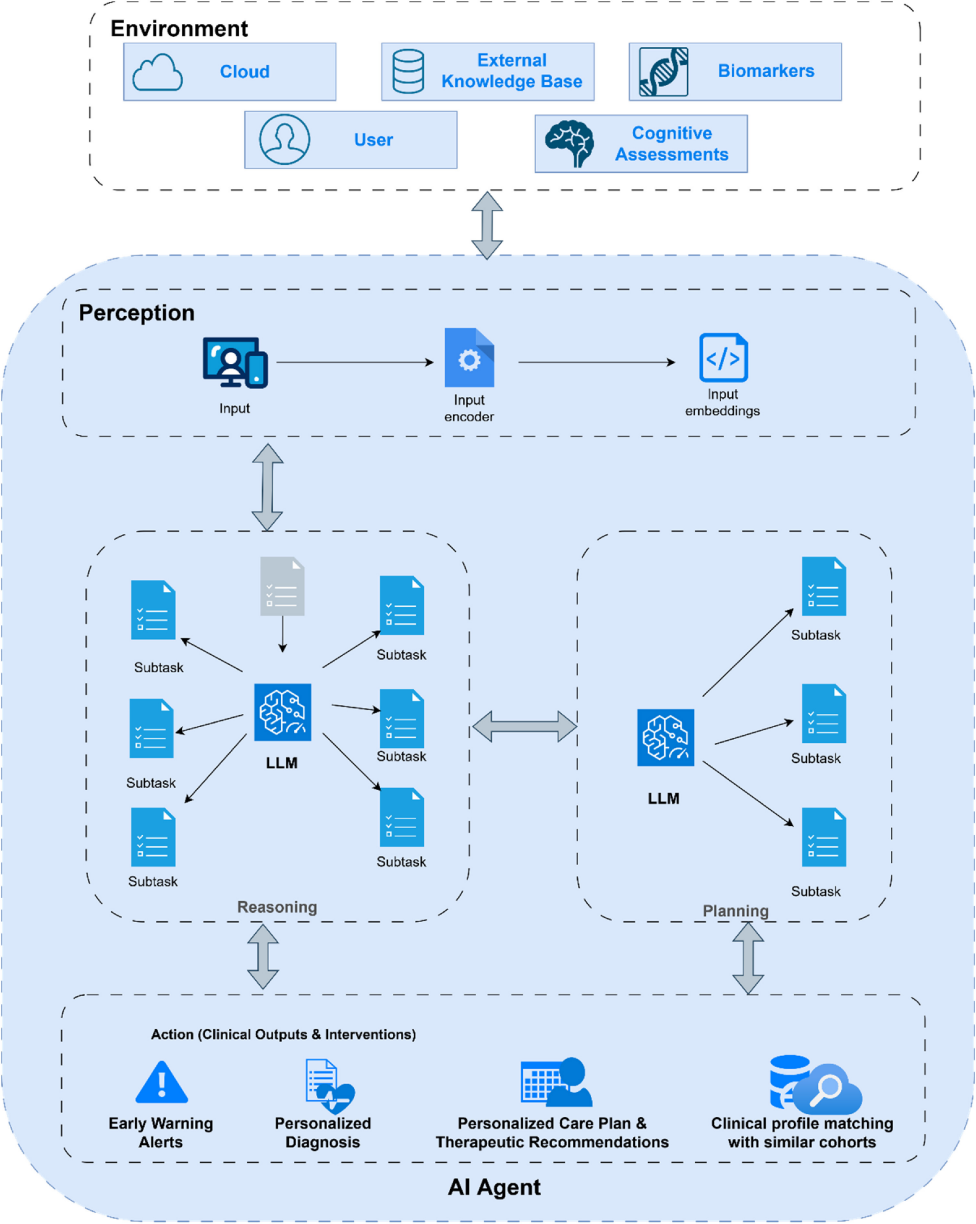
Conceptual framework of an AI agent in Alzheimer’s disease. The agent interacts with environmental data including biomarkers and cognitive assessments through a continuous perception, reasoning, planning, and action cycle. Inputs are encoded into embeddings and processed by large language models to decompose complex tasks. The action layer executes clinical outputs such as early warning alerts, personalized diagnoses, therapeutic recommendations, and clinical profile matching.

The growing importance of agentic AI stems from its capacity to address environments that are complex, uncertain, and data-intensive. This makes it particularly relevant in scientific and medical domains where dynamic systems must be understood and interpreted across multiple layers of biological, clinical, and behavioral information. In the study of neurodegenerative diseases such as Alzheimer’s disease, where disease mechanisms unfold gradually and vary across individuals, agentic systems hold the potential to synthesize vast datasets and extract patterns that evolve over time (Cheng et al., 2024). Their ability to integrate molecular findings, imaging data and cognitive assessments could eventually support the construction of adaptive models capable of anticipating disease progression or identifying subtle early indicators of decline, as Figure 1 depicts. Through this adaptive reasoning, agentic AI offers a conceptual pathway toward a more comprehensive understanding of complex neurobiological processes (Breithaupt et al., 2025).

AI agents can be organized into several main categories depending on their internal architecture and level of autonomy. Reactive agents represent the simplest form, responding directly to stimuli from their environment without storing or reasoning about past experiences. Their lack of memory makes them well-suited for tasks that require immediate reaction or repetitive evaluation of changing conditions (Sapkota et al., 2025). Deliberative agents operate at a higher level, maintaining internal representations of their environment and planning future actions based on predicted outcomes. They reason about alternative possibilities before deciding on a course of action, a feature that makes them potentially useful for modeling long-term biological processes such as disease progression. In neurodegenerative research, such agents can be used to simulate how cellular, molecular, and behavioral factors interact over time, supporting hypothesis generation in studies of brain aging or dementia. Hybrid agents combine both reactive and deliberative mechanisms, enabling immediate responsiveness while retaining strategic, long-term reasoning capabilities (Lanham, 2025). Their architecture allows them to balance real-time data interpretation with broader analytical goals. This flexibility can be valuable in longitudinal biomedical studies, where systems must adapt to new data while maintaining continuity with prior analyses.

Functional distinctions can be identified between goal-based and utility-based agents (Matsumoto et al., 2006). Goal-based agents operate based on predefined objectives, continuously adjusting their decisions to achieve those targets. They are particularly useful in data-driven research pipelines aimed at optimizing model performance or enhancing predictive accuracy. Utility-based agents build upon this principle by evaluating the relative worth of possible outcomes and selecting actions that maximize overall benefit according to specified evaluation criteria. These agents are especially valuable in research settings that demand trade-offs among sensitivity, specificity and interpretability, for example, distinguishing between early and late stages of Alzheimer’s pathology. Learning agents constitute another important category, characterized by their ability to improve through feedback (Axelsson et al., 2022). They refine their decision-making processes as they accumulate experience, which makes them especially relevant in settings where the underlying system evolves or where the available data grow over time. Reinforcement learning, a key approach within this category, enables agents to learn optimal behaviors through iterative exploration and evaluation (Wang F. et al., 2025). Although its full application to neurodegenerative disease research is still emerging, such adaptive learning methods could one day contribute to refining diagnostic models or identifying new therapeutic targets by continuously updating their reasoning as new evidence appears. Finally, multi-agent systems bring together several autonomous entities that cooperate or coordinate to achieve shared objectives (Rother et al., 2025). They can divide complex analytical problems into smaller, specialized tasks and communicate to form a collective intelligence greater than the sum of its parts. While their integration into neuroscience and biomedical research remains largely conceptual, such systems hold promise for managing large-scale datasets that span molecular, imaging, and clinical dimensions, potentially enabling collaborative reasoning across interconnected models.

The expanding development of these various forms of agents illustrates the flexibility and depth of the agentic AI paradigm. While their current use in neurodegenerative research is still in exploratory stages, their theoretical capabilities align closely with the needs of the field (Perry et al., 2024). They offer frameworks for continuous learning, adaptive reasoning, and distributed analysis; qualities that mirror the progressive and multifactorial nature of disorders such as Alzheimer’s disease. As these technologies evolve, the main challenge will be to translate their conceptual promise into practical, transparent, and interpretable tools that can operate responsibly within scientific and clinical research.

## From classical AI tools to AI agents in neurogenerative care

Current AI applications in neurodegenerative disease diagnosis remain narrow, single-task, unimodal systems that function as sophisticated classifiers rather than reasoning applications. These systems showcase narrow intelligence with the application of high-level processing to single, constrained clinical tasks without capacity for generalization or multi-step reasoning (Adler-Milstein et al., 2022). Most models operate on unimodal data performing analysis on neuroimaging biomarkers or conducting clinical assessments in isolation (Shokrpour et al., 2025). While deep learning achieves accuracy greater than 80% on specific classification tasks (Venugopalan et al., 2021), these systems exhibit performance degradation when encountering incomplete data or context-based scenarios that was not part of the training set. Furthermore, no current AI system continually receives feedback about diagnostic performance, limiting capacity for clinical learning and adaption (Ahmed et al., 2023).

AI agents can change this entirely. Unlike traditional machine and deep learning methodologies that map input to output, AI agents execute goal-directed behavior that break high-level complex objectives into smaller subtasks while maintaining probabilistic decision-making. Recent works demonstrate this shift. Google presented the AIME system that uses state-aware reasoning through diagnostic phases that outperforms clinicians in 29 out of 32 evaluations (Saab et al., 2025). MedAgent-Pro introduced hierarchical workflows with disease-level planning and patient-level reasoning (Wang Z. et al., 2025). For neurodegenerative disease, agents can provide guidance that’s impossible to achieve with narrow tools. Instead of producing a simple classification label, an agent can follow a multi-step reasoning process. It may analyze atrophy patterns on MRI, interpret cerebrospinal fluid biomarker ratios, retrieve information from electronic health records about genetic risk factors, and assess the patient’s cognitive trajectory over time. It can also review current diagnostic criteria, generate a probabilistic differential diagnosis, and suggest confirmatory assessments when uncertainty remains high. A 2024 Nature Medicine study involving more than fifty-one thousand participants showed that a multimodal model reached an AUROC of 0.96 in distinguishing ten dementia types, outperforming neurologist-only assessment by more than twenty-six percent (Xue et al., 2024). Yet, even this remains a static classifier. Agentic systems build upon such models with introducing dynamic reasoning which allows them to request additional context for the data provided when confidence drops and track longitudinal disease trajectories to improve understanding over time. Foundation models such as ChatGPT, Claude and even more clinical focused like Med-PaLM2 now provide the intelligence needed for these systems to achieve expert-level performance across a wide range of medical tasks (Singhal et al., 2023, 2025). Evidence from a review of more than four hundred studies shows that multimodal approaches consistently outperform single-modality models by about six percentage points in AUC (Schouten et al., 2025). Agentic architectures could convert those performance gains into real clinical value through the integration of reasoning, memory, and adaptive decision processes that reflect human diagnostic thought.

## Challenges in development and clinical deployment

Recent works in explainable AI in medicine found that the principal barrier to clinical adoption is the black box nature of deep learning models. The authors emphasized that when the reasoning process of a model is not transparent, clinicians are less to rely on its output, particularly in contexts where decisions carry important medical and legal consequences (Muhammad and Bendechache, 2024; Houssein et al., 2025). Clinicians do not need to follow each computational step, but they do require clear and well-grounded explanations that align with accepted medical reasoning and professional accountability. An effective AI agent would present its diagnostic recommendations with narrative justifications that reference key clinical features, supporting evidence, and plausible alternative interpretations. Advances in foundation models now make this possible with enabling the generation of detailed clinical rationales, probabilistic differential diagnoses, and evidence-based references. A systematic review on explainability in clinical AI found that the ethical requirement for transparency depends strongly on context, with low risk screening able to tolerate opaque accuracy, while high risk interventions demand comprehensive and interpretable justification (Blackman and Veerapen, 2025). Holding AI systems to the same standard of explainability expected from human reasoning, rather than to an unattainable level of mathematical clarity, would create a more practical balance between transparency and clinical utility.

Another major challenge in the clinical deployment of AI agents lies in understanding how humans collaborate with these systems and how trust in their outputs is calibrated to effectively support clinical judgment. Experimental evidence in disease diagnostics shows that the introduction of AI support can create a false conflict error, a situation in which clinicians replace correct judgments with incorrect algorithmic outputs (Rosenbacke et al., 2024). Under controlled experimental conditions, physicians demonstrating high baseline diagnostic accuracy exhibited significant performance degradation following the introduction of AI assistance. Specifically, diagnostic accuracy declined from approximately 87% to 77% upon exposure to model-generated recommendations. This decline occurred because clinicians deferred to the system’s suggestions even when their original assessment had been correct. In contrast, clinicians with lower baseline accuracy improved, suggesting that algorithmic guidance can compensate for limited expertise while potentially undermining expert clinical reasoning when over trusted. These findings suggest that the interaction between human expertise and AI recommendations depends not only on model performance but also on the clinician’s perception of the system’s trustworthiness. Overconfidence in algorithmic predictions can lead to errors that would otherwise not occur, whereas excessive skepticism can neutralize potential benefits. This phenomenon, known as trust miscalibration, underscores the importance of designing AI systems that communicate uncertainty, reliability, and contextual limitations. In AD research, similar issues have been observed during the integration of AI models trained on MRI, PET, and multimodal biomarker data, where clinicians may either overestimate model reliability or dismiss valid insights due to insufficient interpretability and lack of transparent reasoning (Mirkin and Albensi, 2023).

## Transition toward agentic tools in clinical practice and future insights

The most practical way to introduce agentic AI systems into healthcare is through targeted augmentation of existing clinical bottlenecks rather than attempting a full-scale transformation of clinical workflows. Applications such as cognitive assessment agents, neuroimaging triage systems, and medication optimization tools represent feasible near-term implementations, it remains unclear whether these narrowly focused deployments will demonstrate sufficient clinical and operational value to justify widespread adoption. Automated screening, for instance, could in theory help reduce clinic wait times, which often exceed six months (Mattke and Hanson, 2024) and even stretch to twelve months (Lam and Mattke, 2021), but its true impact depends on whether such an application genuinely improves patient outcomes or merely redistributes diagnostic delays. In many cases, the limiting factor is not algorithmic performance but the healthcare system’s capacity to respond to an increased number of patients identified for expedited assessment. The main advantage of these domain-specific systems lies in their inherently safe failure modes. Errors in cognitive scoring can be detected through clinician review, prioritization mistakes in imaging typically cause diagnostic delays rather than missed diagnoses, and medication optimization recommendations still require human approval before clinical implementation (Connor et al., 2018). Systems designed primarily for safety and oversight may remain confined to supportive roles and never achieve the degree of autonomy required for substantial improvements in clinical efficiency or systemic scalability.

Coordinated multi agent diagnostic systems that divide the diagnostic process into specialized components such as clinical intake, biomarker integration, literature synthesis, differential diagnosis and treatment recommendation are a promising direction for clinical artificial intelligence (Borkowski and Ben-Ari, 2025). The modularity principle is appealing, as it allows individual components to be updated with advances in medical knowledge and computational methods without rebuilding the entire system. Yet the same flexibility introduces coordination challenges and raises conceptual doubts about whether clinical reasoning can truly be divided into discrete computational stages. In reality, diagnostic reasoning is iterative: clinicians continuously adjust hypotheses as new information and context interact (Olson et al., 2024). Rigidly partitioning subtasks in multi-agent architectures may therefore distort the natural flow of diagnostic thought.

A future step between current diagnostic agents and more autonomous systems may involve integrating aspects that mimic the concept of active inference. Current clinical AI models still operate as passive classifiers that rely on static, pre collected inputs, which limits their ability to emulate the nature of clinical decision making. Future agentic systems should be able to estimate their own uncertainty and determine which additional data would reduce diagnostic uncertainty (Gao et al., 2024). This could involve suggesting a targeted cognitive task, prompting the patient for a specific examination or requesting more data regarding their clinical history. Such targeted information gathering is similar to how clinicians ask specific questions to clarify uncertainty during diagnosis. These systems will still require a human in the loop framework to ensure that any AI initiated data request remains clinically appropriate, feasible and ethically acceptable (Moreno-Sánchez et al., 2026). However, to achieve this level of agentic decision making requires a change in how foundational language models which function as the “brain” of these agents are trained. Current models optimize next token prediction rather than truthfulness or calibration, limiting their ability to identify when their own knowledge is insufficient. Incorporating active inference aspects will likely require training objectives that explicitly model uncertainty to allow the agent to distinguish between medical ambiguity and uncertainty arising from missing information. Without such reasoning, the system cannot reliably decide when it should stop, request additional data, or escalate to clinician oversight.

The emerging paradigm of longitudinal care agents, which continuously synthesize data from wearable sensors, cognitive evaluations, patient-reported outcomes, and administrative health records, marks a transformative yet uncertain frontier in clinical artificial intelligence. These agents could, in theory, detect minimal signs of cognitive or functional decline months before conventional diagnostic thresholds are reached (AlHarkan et al., 2024). They could anticipate clinical inflection points, warn for early interventions, identify candidates for clinical trials, and potentially slow disease progression. However, this faces non-technical challenges that can potentially be more restrictive than the limitations of current algorithms. Continuous AI-driven monitoring depends on infrastructure that in most cases does not yet exist. It requires standardized data exchange protocols across health networks and regulatory frameworks that can guarantee patient autonomy while at the same time allow for algorithmic observation and development and deployment of models that prioritize preventive over reactive care. The assumption that earlier detection will result in better outcomes also warrants careful examination. In neurodegenerative diseases, where disease-modifying therapies remain limited (Yiannopoulou and Papageorgiou, 2020), identifying decline months earlier may contribute primarily prognostic information without offering a meaningful therapeutic benefit. Early detection without effective interventions may cause emotional distress and diminish quality of life, factors not captured by quantitative metrics but crucial for assessing the true value of continuous AI monitoring (Lee et al., 2014; Felekoğlu et al., 2021). Such systems also pose unique risks: longitudinal agents must remain calibrated as patients age and conditions change. Unlike episodic diagnostic tools, continuously running agents can gradually drift from accuracy, with errors accumulating unnoticed. Ensuring long-term oversight and recalibration is therefore essential, yet remains an unresolved challenge for safe deployment.

As the field advances, several foundational questions warrant continued deliberation. Should AI agents aim for superhuman performance based on non-human reasoning, or should validation prioritize interpretable models aligned with human cognition? Current evaluation standards assume equivalence with expert performance, yet human experts exhibit systematic biases, anchoring on initial impressions (Ly et al., 2023), overemphasizing recent salient cases, and neglecting base rates (Saposnik et al., 2016). Agents free from these cognitive constraints might achieve superior outcomes through reasoning patterns that diverge from human approaches. The question becomes whether we accept agents outperforming humans through non-human reasoning and recognize that the goal is improved patient outcomes rather than replicating human thought processes. What performance threshold justifies transitioning agents from advisory to autonomous roles? Human physicians maintain autonomous authority despite diagnostic error rates of 10%–15% (Graber, 2013). Applying higher standards to AI than humans could result in status quo biases while delaying patient access to superior diagnostic tools. Risk-stratified governance distinguishing advisory recommendations with mandatory human confirmation from default-accept recommendations with override capability to autonomous actions with retrospective review enables proportionate oversight matching autonomy levels to clinical risk.

Agentic AI systems hold genuine potential to address critical limitations in neurodegenerative disease diagnosis through integrating multimodal data, maintaining longitudinal surveillance, and reducing clinician cognitive burden. However, the transition from current narrow AI tools to clinically deployable autonomous systems is far from straightforward. It involves navigating unresolved technical limitations, regulatory uncertainty, difficulties in workflow integration, and fundamental questions concerning the appropriate scope of algorithmic participation in medical decision-making (Hosseini and Seilani, 2025). A balanced approach grounded in cautious optimism is therefore warranted. Rather than assuming inevitable transformation, progress should emphasize implementation that produces real-world evidence, systematic monitoring of failure modes, and adaptive regulatory frameworks that both enable innovation and protect patient safety.

## Conclusion

Agentic AI represents a new phase of intelligent systems, characterized by reasoning, adaptability, and purposeful action. Although its application in neurodegenerative research is still early, its theoretical capabilities align closely with the needs of Alzheimer’s disease research. By enabling dynamic, context-aware analysis across imaging, genomics, cognitive, and behavioral data, agentic AI offers unprecedented potential to track disease progression and identify therapeutic targets. As a future perspective, these agents may gradually move beyond passive analysis toward models that can detect when their own information is incomplete and initiate targeted data requests to reduce uncertainty. This shift would push agentic systems closer to real clinical reasoning while still keeping clinicians in control. Ensuring ethical and patient centered deployment through close collaboration between technical and clinical teams remains essential, shifting the focus from whether AI will reshape care to how it can meaningfully support clinical expertise and patient outcomes.

## Data Availability

The original contributions presented in this study are included in this article/supplementary material, further inquiries can be directed to the corresponding author.

## References

[B1] Adler-Milstein J. Aggarwal N. Ahmed M. Castner J. Evans B. J. Gonzalez A. A. (2022). Meeting the moment: addressing barriers and facilitating clinical adoption of artificial intelligence in medical diagnosis. *NAM Perspect* 2022:10.31478/202209c. 10.31478/202209c. 36713769 PMC9875857

[B2] Agostinho D. Simões M. Castelo-Branco M. (2024). Predicting conversion from mild cognitive impairment to Alzheimer’s disease: a multimodal approach. *Brain Commun*. 6:fcae208. 10.1093/braincomms/fcae208 38961871 PMC11220508

[B3] Ahmed M. I. Spooner B. Isherwood J. Lane M. Orrock E. Dennison A. (2023). A systematic review of the barriers to the implementation of artificial intelligence in healthcare. *Cureus* 15:e46454. 10.7759/cureus.46454 37927664 PMC10623210

[B4] Ai M. Liu Y. Liu D. Yan C. Wang X. Chen X. (2025). Research progress in predicting the conversion from mild cognitive impairment to Alzheimer’s disease via multimodal MRI and artificial intelligence. *Front. Neurol*. 16:1596632. 10.3389/fneur.2025.1596632 40529431 PMC12171368

[B5] AlHarkan K. Sultana N. Al Mulhim N. AlAbdulKader A. M. Alsafwani N. Barnawi M. (2024). Artificial intelligence approaches for early detection of neurocognitive disorders among older adults. *Front. Comput. Neurosci.* 18:1307305. 10.3389/fncom.2024.1307305 38444404 PMC10913197

[B6] Alzheimer’s Disease International. (2024). *World-Alzheimer-Report-2024.* Available: https://www.alzint.org/u/World-Alzheimer-Report-2024.pdf [Accessed October 21, 2025].

[B7] Arikan F. B. Cetintas D. Aksoy A. Yildirim M. (2025). A deep learning approach to Alzheimer’s Diagnosis Using EEG Data: dual-attention and Optuna-Optimized SVM. *Biomedicines* 13:2017. 10.3390/biomedicines13082017 40868268 PMC12383294

[B8] Axelsson A. Buschmeier H. Skantze G. (2022). Modeling feedback in interaction with conversational agents—a review. *Front. Comput. Sci.* 4:744574. 10.3389/fcomp.2022.744574

[B9] Blackman J. Veerapen R. (2025). On the practical, ethical, and legal necessity of clinical Artificial Intelligence explainability: an examination of key arguments. *BMC Med. Inform. Decis. Mak*. 25:111. 10.1186/s12911-025-02891-2 40045339 PMC11881432

[B10] Borkowski A. A. Ben-Ari A. (2025). Multiagent AI systems in health care: envisioning next-generation intelligence. *Fed. Pract*. 42 188–194. 10.12788/fp.0589 40831649 PMC12360800

[B11] Breithaupt A. G. Tang A. Miller B. L. Pinheiro-Chagas P. (2025). Integrating generative artificial intelligence in ADRD: a framework for streamlining diagnosis and care in neurodegenerative diseases. *arXiv [Preprint].* 10.48550/arXiv.2502.06842

[B12] Chen P. Zhao K. Zhang H. Wei Y. Wang P. Wang D. (2023). Altered global signal topography in Alzheimer’s disease. *EBioMedicine* 89:104455. 10.1016/j.ebiom.2023.104455 36758481 PMC9941064

[B13] Cheng F. Wang F. Tang J. Zhou Y. Fu Z. Zhang P. (2024). Artificial intelligence and open science in discovery of disease-modifying medicines for Alzheimer’s disease. *Cell Rep. Med*. 5:101379. 10.1016/j.xcrm.2023.101379 38382465 PMC10897520

[B14] Connor D. J. Jenkins C. W. Carpenter D. Crean R. Perera P. (2018). Detection of rater errors on cognitive instruments in a clinical trial setting. *J. Prev. Alzheimers Dis*. 5 188–196. 10.14283/jpad.2018.20 29972212 PMC12280823

[B15] DeTure M. A. Dickson D. W. (2019). The neuropathological diagnosis of Alzheimer’s disease. *Mol. Neurodegener*. 14:32. 10.1186/s13024-019-0333-5 31375134 PMC6679484

[B16] Felekoğlu E. Özalevli S. Yakut H. Aktan R. Yener G. (2021). Investigation of the factors affecting quality of life in patients with mild to moderate Alzheimer’s disease in terms of patients and caregivers. *Medicina* 57:1067. 10.3390/medicina57101067 34684104 PMC8538831

[B17] Gao S. Fang A. Huang Y. Giunchiglia V. Noori A. Schwarz J. R. (2024). Empowering biomedical discovery with AI agents. *Cell* 187 6125–6151. 10.1016/j.cell.2024.09.022 39486399

[B18] Graber M. L. (2013). The incidence of diagnostic error in medicine. *BMJ Qual. Saf.* 22 (Suppl. 2), ii21–ii27. 10.1136/bmjqs-2012-001615 23771902 PMC3786666

[B19] Hosseini S. Seilani H. (2025). The role of agentic ai in shaping a smart future: a systematic review. *Array* 26:100399. 10.1016/j.array.2025.100399

[B20] Houssein E. H. Gamal A. M. Younis E. M. Mohamed E. (2025). Explainable artificial intelligence for medical imaging systems using deep learning: a comprehensive review. *Cluster Comput.* 28:469. 10.1007/s10586-025-05281-5

[B21] Huang K. (2025). *Agentic AI: Theories and Practices. in Progress in IS.* Cham: Springer Nature Switzerland, 10.1007/978-3-031-90026-6

[B22] Jasodanand V. H. Kowshik S. S. Puducheri S. Romano M. F. Xu L. Au R. (2025). AI-driven fusion of multimodal data for Alzheimer’s disease biomarker assessment. *Nat. Commun*. 16:7407. 10.1038/s41467-025-62590-4 40789853 PMC12339743

[B23] Judge C. S. Krewer F. O’Donnell M. J. Kiely L. Sexton D. Taylor G. W. (2024). Multimodal artificial intelligence in medicine. *Kidney360* 5 1771–1779. 10.34067/KID.0000000000000556 39167446 PMC12282626

[B24] Kale M. Wankhede N. Pawar R. Ballal S. Kumawat R. Goswami M. (2024). AI-driven innovations in Alzheimer’s disease: integrating early diagnosis, personalized treatment, and prognostic modelling. *Ageing Res. Rev*. 101:102497. 10.1016/j.arr.2024.102497 39293530

[B25] Lam J. Mattke S. (2021). Memory care approaches to better leverage capacity of dementia specialists: a narrative synthesis. *Neurodegener. Dis. Manag*. 11 239–250. 10.2217/nmt-2020-0038 33966489

[B26] Lanham M. (2025). *AI agents in Action.* Shelter Island, NY: Manning Publications.

[B27] Lee S. M. Roen K. Thornton A. (2014). The psychological impact of a diagnosis of Alzheimer’s disease. *Dement. Lond. Engl.* 13 289–305. 10.1177/1471301213497080 24339103

[B28] Lee T. Lee H. (2020). Prediction of Alzheimer’s disease using blood gene expression data. *Sci. Rep*. 10:3485. 10.1038/s41598-020-60595-1 32103140 PMC7044318

[B29] Luo G. Zhang J. Song Z. Wang Y. Wang X. Qu H. (2023). Effectiveness of non-pharmacological therapies on cognitive function in patients with dementia-A network meta-analysis of randomized controlled trials. *Front. Aging Neurosci.* 15:1131744. 10.3389/fnagi.2023.1131744 36967820 PMC10035791

[B30] Ly D. P. Shekelle P. G. Song Z. (2023). Evidence for anchoring bias during physician decision-making. *JAMA Intern. Med*. 183 818–823. 10.1001/jamainternmed.2023.2366 37358843 PMC10294014

[B31] Mahmud T. Barua K. Habiba S. U. Sharmen N. Hossain M. S. Andersson K. (2024). An Explainable AI Paradigm for Alzheimer’s diagnosis using deep transfer learning. *Diagnostics* 14:345. 10.3390/diagnostics14030345 38337861 PMC10855149

[B32] Matsumoto K. Matsumoto M. Abe H. (2006). Goal-based action selection and utility-based action bias. *Neural Netw.* 19 1315–1320. 10.1016/j.neunet.2006.05.036 16942861

[B33] Mattke S. Hanson M. (2024). Expected wait times for access to a disease-modifying Alzheimer’s treatment in the United States. *Alzheimers Dement*. 18 1071–1074. 10.1002/alz.12470 34569686

[B34] Mirkin S. Albensi B. C. (2023). Should artificial intelligence be used in conjunction with Neuroimaging in the diagnosis of Alzheimer’s disease? *Front. Aging Neurosci*. 15:1094233. 10.3389/fnagi.2023.1094233 37187577 PMC10177660

[B35] Moreno-Sánchez P. A. Del Ser J. van Gils M. Hernesniemi J. (2026). A design framework for operationalizing trustworthy artificial intelligence in healthcare: requirements, tradeoffs and challenges for its clinical adoption. *Informat. Fusion* 127:103812. 10.1016/j.inffus.2025.103812

[B36] Muhammad D. Bendechache M. (2024). Unveiling the black box: a systematic review of Explainable Artificial Intelligence in medical image analysis. *Comput. Struct. Biotechnol. J*. 24 542–560. 10.1016/j.csbj.2024.08.005 39252818 PMC11382209

[B37] Ó hAnrachtaigh É Brown G. Beck A. Conway R. Jones H. Angelakis I. (2024). Transdiagnostic psychological interventions for symptoms of common mental disorders delivered by non-specialist providers in low- and middle-income countries: a systematic review and meta-analysis. *Depress Anxiety* 2024:5037662. 10.1155/2024/5037662 40226747 PMC11921846

[B38] Olson A. Kämmer J. E. Taher A. Johnston R. Yang Q. Mondoux S. (2024). The inseparability of context and clinical reasoning. *J. Eval. Clin. Pract*. 30 533–538. 10.1111/jep.13969 38300231

[B39] Perry N. Sun C. Munro M. Boulton K. A. Guastella A. J. (2024). AI technology to support adaptive functioning in neurodevelopmental conditions in everyday environments: a systematic review. *NPJ Digit Med*. 7:370. 10.1038/s41746-024-01355-7 39702672 PMC11659516

[B40] Qi W. Zhu X. Wang B. Shi Y. Dong C. Shen S. (2025). Alzheimer’s disease digital biomarkers multidimensional landscape and AI model scoping review. *NPJ Digit Med*. 8:366. 10.1038/s41746-025-01640-z 40523935 PMC12170881

[B41] Rosenbacke R. Melhus Å Stuckler D. (2024). False conflict and false confirmation errors are crucial components of AI accuracy in medical decision making. *Nat. Commun*. 15:6896. 10.1038/s41467-024-50952-3 39138179 PMC11322186

[B42] Rother D. Pajarinen J. Peters J. Weisswange T. H. (2025). Open-ended coordination for multi-agent systems using modular open policies. *Autonom. Agents Multi-Agent Syst.* 39 1–28. 10.1007/s10458-025-09723-7

[B43] Rudin C. (2019). Stop explaining black box machine learning models for high stakes decisions and use interpretable models instead. *Nat. Mach. Intell*. 1 206–215. 10.1038/s42256-019-0048-x 35603010 PMC9122117

[B44] Saab K. Freyberg J. Park C. Strother T. Cheng Y. Weng W. H. (2025). Advancing conversational diagnostic ai with multimodal reasoning. *arXiv [Preprint]* 10.48550/arXiv.2505.04653 PMC13190284 42135531

[B45] Sapkota R. Roumeliotis K. I. Karkee M. (2025). Ai agents vs. agentic ai: A conceptual taxonomy, applications and challenges. *arXiv [Preprint]* 10.1016/j.inffus.2025.103599

[B46] Saposnik G. Redelmeier D. Ruff C. C. Tobler P. N. (2016). Cognitive biases associated with medical decisions: a systematic review. *BMC Med. Inform. Decis. Mak*. 16:138. 10.1186/s12911-016-0377-1 27809908 PMC5093937

[B47] Schouten D. Nicoletti G. Dille B. Chia C. Vendittelli P. Schuurmans M. (2025). Navigating the landscape of multimodal AI in medicine: a scoping review on technical challenges and clinical applications. *Med. Image Anal.* 105:103621. 10.1016/j.media.2025.103621 40482561

[B48] Shokrpour S. MoghadamFarid A. Bazzaz Abkenar S. Haghi Kashani M. Akbari M. Sarvizadeh M. (2025). Machine learning for Parkinson’s disease: a comprehensive review of datasets, algorithms, and challenges. *NPJ Parkinsons Dis*. 11:187. 10.1038/s41531-025-01025-9 40595773 PMC12217022

[B49] Singhal K. Azizi S. Tu T. Mahdavi S. S. Wei J. Chung H. W. (2023). Large language models encode clinical knowledge. *Nature* 620 172–180. 10.1038/s41586-023-06291-2 37438534 PMC10396962

[B50] Singhal K. Tu T. Gottweis J. Sayres R. Wulczyn E. Amin M. (2025). Toward expert-level medical question answering with large language models. *Nat. Med*. 31 943–950. 10.1038/s41591-024-03423-7 39779926 PMC11922739

[B51] Tang A. S. Rankin K. P. Cerono G. Miramontes S. Mills H. Roger J. (2024). Leveraging electronic health records and knowledge networks for Alzheimer’s disease prediction and sex-specific biological insights. *Nat. Aging* 4 379–395. 10.1038/s43587-024-00573-8 38383858 PMC10950787

[B52] van Oostveen W. M. de Lange E. C. M. (2021). Imaging techniques in Alzheimer’s disease: a review of applications in early diagnosis and longitudinal monitoring. *Int. J. Mol. Sci*. 22:2110. 10.3390/ijms22042110 33672696 PMC7924338

[B53] Venugopalan J. Tong L. Hassanzadeh H. R. Wang M. D. (2021). Multimodal deep learning models for early detection of Alzheimer’s disease stage. *Sci. Rep*. 11:3254. 10.1038/s41598-020-74399-w 33547343 PMC7864942

[B54] Wang F. Li S. Niu S. Yang H. Li X. Deng X. (2025). A Survey on recent advances in reinforcement learning for intelligent investment decision-making optimization. *Expert Syst. Appl.* 282:127540. 10.1016/j.eswa.2025.127540

[B55] Wang S. Wang Y. Xu F. H. Tian X. Fredericks C. A. Shen L. (2024). Sex-specific topological structure associated with dementia via latent space estimation. *Alzheimers Dement*. 20 8387–8401. 10.1002/alz.14266 39530632 PMC11667551

[B56] Wang Z. Wu J. Cai L. Low C. H. Yang X. Li Q. (2025). MedAgent-Pro: towards evidence-based multi-modal medical diagnosis via reasoning agentic workflow. *arXiv [Preprint]* 10.48550/arXiv.2503.18968

[B57] Winchester L. M. Harshfield E. L. Shi L. Badhwar A. Khleifat A. A. Clarke N. (2023). Artificial intelligence for biomarker discovery in Alzheimer’s disease and dementia. *Alzheimers Dement*. 19 5860–5871. 10.1002/alz.13390 37654029 PMC10840606

[B58] Xu H. Shuttleworth K. M. J. (2024). Medical artificial intelligence and the black box problem: a view based on the ethical principle of ‘do no harm. *Intell. Med.* 4 52–57. 10.1016/j.imed.2023.08.001

[B59] Xue C. Kowshik S. S. Lteif D. Puducheri S. Jasodanand V. H. Zhou O. T. (2024). AI-based differential diagnosis of dementia etiologies on multimodal data. *Nat. Med*. 30 2977–2989. 10.1038/s41591-024-03118-z 38965435 PMC11485262

[B60] Yiannopoulou K. G. Papageorgiou S. G. (2020). Current and future treatments in Alzheimer disease: an update. *J. Cent. Nerv. Syst. Dis*. 12:1179573520907397. 10.1177/1179573520907397 32165850 PMC7050025

